# Machine learning and EEG can classify passive viewing of discrete categories of visual stimuli but not the observation of pain

**DOI:** 10.1186/s12868-023-00819-y

**Published:** 2023-09-15

**Authors:** Tyler Mari, Jessica Henderson, S. Hasan Ali, Danielle Hewitt, Christopher Brown, Andrej Stancak, Nicholas Fallon

**Affiliations:** Department of Psychology, Institute of Population Health, University of Liverpool, 2.21 Eleanor Rathbone Building, Bedford Street South, Liverpool, L69 7ZA UK

**Keywords:** Empathy, Electroencephalography, N170, Event-related potential, External validation

## Abstract

Previous studies have demonstrated the potential of machine learning (ML) in classifying physical pain from non-pain states using electroencephalographic (EEG) data. However, the application of ML to EEG data to categorise the observation of pain versus non-pain images of human facial expressions or scenes depicting pain being inflicted has not been explored. The present study aimed to address this by training Random Forest (RF) models on cortical event-related potentials (ERPs) recorded while participants passively viewed faces displaying either pain or neutral expressions, as well as action scenes depicting pain or matched non-pain (neutral) scenarios. Ninety-one participants were recruited across three samples, which included a model development group (n = 40) and a cross-subject validation group (n = 51). Additionally, 25 participants from the model development group completed a second experimental session, providing a within-subject temporal validation sample. The analysis of ERPs revealed an enhanced N170 component in response to faces compared to action scenes. Moreover, an increased late positive potential (LPP) was observed during the viewing of pain scenes compared to neutral scenes. Additionally, an enhanced P3 response was found when participants viewed faces displaying pain expressions compared to neutral expressions. Subsequently, three RF models were developed to classify images into faces and scenes, neutral and pain scenes, and neutral and pain expressions. The RF model achieved classification accuracies of 75%, 64%, and 69% for cross-validation, cross-subject, and within-subject classifications, respectively, along with reasonably calibrated predictions for the classification of face versus scene images. However, the RF model was unable to classify pain versus neutral stimuli above chance levels when presented with subsequent tasks involving images from either category. These results expand upon previous findings by externally validating the use of ML in classifying ERPs related to different categories of visual images, namely faces and scenes. The results also indicate the limitations of ML in distinguishing pain and non-pain connotations using ERP responses to the passive viewing of visually similar images.

## Introduction

Machine learning (ML) and EEG have demonstrated promise for predicting discrete categories of visual stimuli (e.g., objects, scenes, faces etc.) [1–7], subjective pain intensity in response to physical pain [8–10], and response to pharmaceutical intervention [11–13], to name but a few. Research from our group previously demonstrated that high and low pain stimuli can be predicted with approximately 70% accuracy using time–frequency analysis of EEG features distributed across the scalp [9]. However, the effectiveness of ML and EEG for the classification of human facial expressions and scenes depicting pain and non-pain conditions has yet to be explored. This is despite a wealth of research demonstrating the importance of neurobiological empathic responses to observed pain, which has particular relevance to clinical, physiological, and societal domains [14–17]. For example, elucidating the neurobiology of empathy is important for understanding the development of empathy and for clinical conditions where empathy is reduced or absent (e.g., autism) [18–20]. Moreover, from a societal perspective, understanding the neurobiology of empathy may support areas such as medical education [21]. Therefore, this study aimed to address this gap by developing ML models using single-trial EEG responses during the passive observation of both facial expressions and action scenes depicting neutral and painful conditions.

Traditional ERP research studies exploring empathic responses to the observation of pain demonstrate differences in ERP amplitudes, which may enable accurate ML classification at the single-trial level. A meta-analysis of up to 36 studies demonstrated an enhanced P3 and late positive potential (LPP) during pain observation, with the maximal effect observed at central-parietal sites [22]. Previous research by our lab demonstrated that images depicting pain scenes elicited an enhanced LPP over central-parietal regions compared to situation-matched neutral images in both healthy people and a chronic pain population [23]. Therefore, single-trial EEG responses over central-parietal electrode sites may be an important candidate feature for the ML algorithm.

In addition to classifying EEG responses to images depicting neutral and pain conditions, we also aimed to externally validate ML for the classification of single-trial neural responses to broad categories of visual stimuli (faces versus scenes) regardless of the pain component, which to the best of our knowledge has yet to be attempted. Here, the N170 component may be the most informative feature for classification. The N170 component is an early negative waveform deflection which is maximally observed over occipitotemporal regions between 140 and 200 ms after stimulus onset, peaking at approximately 170 ms, which is enhanced during the observation of faces [24, 25]. The N170 is maximal when viewing faces and is attenuated or missing in response to other stimulus categories [25, 26]. The N170 has been reliably reproduced in stationary and mobile EEG experiments [24–30]. Additionally, the vertex positive potential (VPP), which is a large positive potential across frontal-central regions peaking between 140 and 180 ms, is observed after the presentation of a face stimulus [24, 31, 32]. Given the similarity in the characteristics of the N170 and VPP, the evidence suggests that both components originate from the same neural dipole [33, 34]. Therefore, neural responses located over occipitotemporal and frontal-central regions may enable accurate classification of face versus scene images.

Indeed, previous research has successfully combined EEG and ML to classify neural responses to visual stimuli including faces, objects, and scenes. A support vector machine (SVM) trained on EEG components over occipital electrodes has successfully classified the presence of visual objects in 7 subjects; achieving a cross-validated accuracy and AUC of 87% and 0.7, respectively [1]. Additionally, research has demonstrated that neural networks could successfully classify 40 image classes from the ImageNet database (e.g., animals, objects, food) with an average accuracy of 90.16% using EEG recorded from 6 subjects [2]. Further research exhibits comparable results in decoding neural responses to objects, scenes, human and animal bodies and faces [3–6]. Finally, an attention-based convolutional bidirectional long short-term memory network has been developed to classify EEG responses to familiar and unfamiliar faces [7]. Using time–frequency features from pre-frontal, frontal, and temporal regions, the authors classified familiar and unfamiliar faces with an accuracy of 91.34%. Therefore, the literature suggests that EEG and ML can potentially be used to successfully decode brain responses to categories of visual stimuli.

Despite promising results, the field is not without significant limitations. ML research is often insufficiently validated, with only internal validation methods used to evaluate models. This potentially leads to inflated performance estimates, overfitting and un-generalisable models [35–37]. Therefore, ML models should be evaluated using data independent of model development [38]. One such approach is external validation, whereby ML performance is assessed using novel data obtained from other cohorts, facilities, and repositories or collected from a different location (geographical), time (temporal) or experimental paradigm [37, 39]. Research has demonstrated reduced performance on external validation datasets [9, 40, 41]. Due to the omission of external validation, it is challenging to reasonably interpret the generalisability of existing research, as the results are potentially inflated.

The present study aimed to externally validate ML and EEG for visual stimuli decoding both across and within subjects for the first time. Firstly, we trained a Random Forest (RF) model on EEG features to classify data into either faces or scenes. Moreover, we developed two further RF models to classify EEG data into either neutral or pain classes for both scenes and faces respectively. All models were externally validated using two separate samples: cross-subject which consisted of a new cohort, and within-subject which consisted of participants from the model development sample who were recruited for a second experimental session at a later time (temporal validation). We hypothesised that the RF model would classify visual stimuli with an accuracy significantly greater than the chance level (≈ 50%) for each classification task: (1) faces—scenes, (2) scenes: neutral—pain, and (3) faces: neutral—pain for both external validation samples.

## Methods

### Participants

A total of three samples, consisting of 116 EEG sessions, were collected for this study. Forty participants (22 female; 7 left-handed) aged between 18 and 52 (Mean = 27.70 years, standard deviation {SD} = 7.43) years were recruited for sample one (model development sample/cross-validation). Sample two (cross-subject validation) consisted of 51 participants (34 female; 6 left-handed) aged between 19 and 60 (Mean = 27.63 years, SD = 9.65), whilst sample three consisted of 25 participants aged between 21 and 53 (14 female; 4 left-handed; Mean = 28.96 years, SD = 8.01). Twenty-five participants from sample one completed a second experimental session a minimum of 12 weeks after their first session (Mean = 108.68 days, SD = 10.92). This cohort represented a temporal within-subject validation sample (sample three) for the ML analysis. We aimed to recruit a large sample, particularly for external validation, to provide robust estimates of model generalisability, as small external validation datasets can also provide imprecise estimates of model discrimination and calibration [42]. Participants provided written informed consent before participation and all methods were conducted in compliance with the Declaration of Helsinki. The study received ethical approval from the University of Liverpool Health and Life Sciences Research Ethics Committee. Eligibility criteria included: at least 18 years old, normal, or corrected-to-normal vision, no acute pain at the time of participating, no history of chronic pain, and no neurological conditions. Participants were compensated with a total of £40 for time and travel expenses. The raw data is available on reasonable request.

## Materials

### Pain faces

**Fig. 1:**
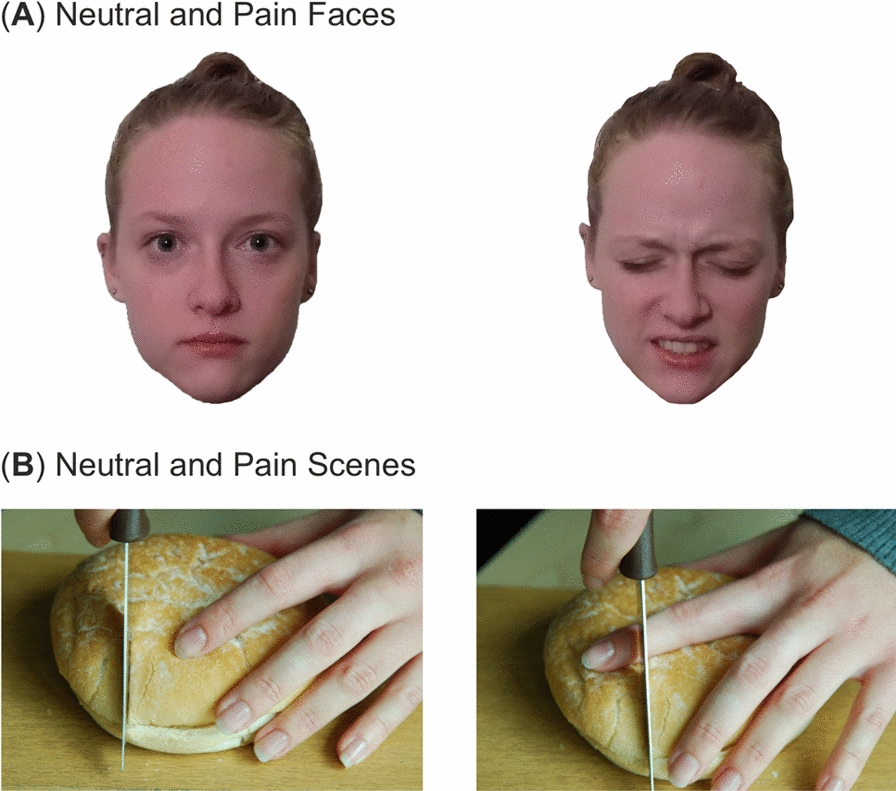
**A** Example of neutral and pain face stimuli from the Delaware Pain Database [44]. **B** Example neutral and pain scene stimuli

In the present study, we employed a passive viewing paradigm where participants were required to observe a series of visual stimuli but were not required to respond. This differs from a free viewing task, as participants were requested to pay attention to the image, which imposes a task and is arguably not truly free viewing [43]. Here, a 2 × 2 factorial design was used in this study: faces (expressions) and scenes, each with two levels, namely neutral and pain. The neutral and pain faces were selected from the Delaware Pain Database [44]. The Delaware Pain Database is an image database that contains photographs of the faces of individuals who are displaying a painful expression (e.g., grimacing) and matched neutral controls. We selected a total of 56 faces (28 painful and 28 matched neutral images). The faces were selected using several criteria. Firstly, we aimed to broadly recreate the ethnicity and gender distribution of the UK to provide representative stimuli. A total of 22 white subjects (80%) consisting of 11 males and females, 3 Asian subjects (10%) including 2 males and 1 female and 3 black subjects (10%) consisting of 1 male and 2 females were selected, which broadly matched the racial distribution of the UK [45]. Within the individual categories (e.g., white males) the images with the highest pain rating were selected, providing pain was listed as the dominant emotion. The 28 neutral images were selected as the matched version (e.g., same subject) of the pain expressions. Face images were approximately 1382 × 925 in size. Figure 1A demonstrates an example of neutral and pain expressions.

### Pain scenes

Additionally, still, photograph images of action scenes depicting pain or matched non-pain scenarios (hereinafter referred to as neutral or pain scenes) were employed in the present study. The pain scene images consisted of 28 images depicting either hands or feet in scenarios that elicit pain. For example, images of a knife cutting through bread in a way that would endanger the finger (e.g., placed under the knife). Twenty-eight matched neutral scenes, which replicate the scene but did not demonstrate pain, were also used. For example, the image depicted a knife cutting through bread without endangering the finger (e.g., the finger not placed under the knife). The same distribution of ethnicities implemented in the facial expression images was applied to the pain scene images. The images were selected from a larger internal pool of photographs depending on their pain rating. A small pilot study was conducted (n = 5) to rate each of the images in terms of pain intensity. The images that elicited the highest average pain rating in the pilot study were selected for the final experiment. The images used in this study are similar to previous research [23, 46–49]. Pain scene images were 774 × 518 in size. Figure 1B demonstrates examples of neutral and pain scene images used in this study.

### Procedure

Participants attended the EEG laboratory at the University of Liverpool between June and October 2022. Following the fitting of the EEG cap, participants were seated inside a Faraday cage 1 m away from a 23-inch 1080p LCD monitor. The experimenter verbally explained the passive viewing task and the participants’ questions were answered. During this time, participants were requested to pay attention to the images and minimise movement during trials. The experiment consisted of a total of 336 trials, split into three blocks of 112 stimuli. Within each block, 28 stimuli for each of the four conditions were presented. Each block lasted 6 min and was separated by approximately 15-min periods. During the block intervals, electrode impedances were checked, and additional saline solution was applied as required.

Each trial was initiated with a 2-s rest interval, where participants were shown a blank grey screen. Following the rest period, a colour photograph, that was randomly selected, was displayed for 1 s. Subsequently, the image disappeared, and the 2-s rest interval occurred before the presentation of the next image. This was repeated until all 112 images had been presented.

Following the completion of all blocks, the EEG cap was removed, and a subjective rating block was completed. Here, participants were informed that they were required to rate their perceived pain intensity of the images on a 0–100 scale with 0 reflecting no pain and 100 reflecting extreme pain. The rating scale included vertical bars denoting increments of 10. During the rating period, participants were presented with an image positioned above the rating scale and were required to rate the image by clicking the scale with the mouse in their right hand. The presentation of the images was randomised, and for each image, an infinite response time was employed. Once the participant had successfully rated the image, the screen was cleared, and the next image and scale were presented 100 ms later. Following this, participants completed the pain catastrophizing scale (PCS) [50] and were subsequently debriefed and compensated for their time and expenses.

### EEG acquisition

Continuous EEG recordings were acquired using a 129-channel EGI System (Electrical Geodesic Inc., EGI, now Magstim EGI, Eugene, Oregon, USA) and a sponge-based Geodesic sensor net. The net was positioned with respect to three anatomical landmarks: two pre-auricular points and the nasion. Throughout the experiment, electrode-to-skin impedances were maintained below 50 kΩ. A recording bandpass filter was applied between 0.001 and 200 Hz and the sampling rate was set at 1000 Hz. Cz was used as the reference electrode.

### EEG data analysis

The data were pre-processed using the Harvard Automated Processing Pipeline for Electroencephalography (HAPPE version 3) [51]. Firstly, low-pass and high-pass filters were applied to the data at 45 and 0.1 Hz, respectively. Secondly, the data were downsampled to 500 Hz and re-referenced using the common average approach [52]. Moreover, bad channel detection and interpolation were performed, and data contaminated by artefacts (e.g., oculographic) underwent wavelet thresholding (soft margin) to separate artefact and neural data. The data were then segmented into epochs of − 200 ms to 800 ms relative to stimulus onset (500 total time points) and baseline corrected (− 200 ms to 0 ms). Automated epoch rejection was then performed based on segment amplitude and similarity criteria. The thresholds were set at minimum and maximum segment amplitude of − 150 and 150, respectively in line with HAPPE recommendations [51]. The number of trials (mean ± SD) retained after automated trial rejection was 60.18 ± 8.44 (72% of total trials) for neutral scenes, 61.23 ± 6.19 (73%) for pain scenes, 62.93 ± 7.87 (75%) for neutral faces, and 62.15 ± 6.90 (74%) for pain faces, in sample one. In sample two, the mean number of trials remaining was 61.88 ± 5.14 (74%) for neutral scenes, 61.78 ± 6.22 (74%) for pain scenes, 62.63 ± 4.81 (75%) for neutral faces, and 62.27 ± 5.19 (74%) for pain faces. Finally, for sample three, the remaining number of trials was 62.76 ± 6.36 (75%) for neutral scenes, 60.20 ± 5.89 (72%) for pain scenes, 63.80 ± 5.97 (76%) for neutral faces, and 64.08 ± 6.49 (76%) for pain faces. Following pre-processing, the ERPs were analysed in MATLAB 2020b (The MathWorks, Inc., Natick, Massachusetts, USA) and EEGLAB 2021.1 [53]. Multiple comparisons were accounted for using the false discovery rate (FDR) method. A minimum window width of 10 ms was implemented to assess significant differences between the ERP waveforms.

### Machine learning procedure

Following EEG pre-processing, the data were prepared for ML analysis. Each of the datasets (model development, cross-subject, and within-subject validation sample) were processed independently to prevent data leakage which could bias the external validation procedure [54]. Candidate features were calculated from single-trial ERP waveforms. A total of 18 candidate features, which primarily represented descriptive statistics of the ERP waveform, were calculated for each trial between 0 and 800 ms relative to stimulus onset. The features consisted of the mean, mode, median, minimum, maximum, standard deviation, root mean squared, variance, skewness, kurtosis, absolute mean, Shannon entropy, log energy entropy, range, mean squared, number of peaks, number of troughs, and the ratio between peaks and troughs. The features calculated in this study are comparable to previous research, both by our lab and external groups [9, 55–58]. The 18 features were calculated using MATLAB functions, where possible, and were computed for each of the 129 electrodes, resulting in 2322 candidate features.

Single-trial EEG is significantly impacted by noise and variability [59–61]. In line with our previous research, outlier feature values, defined as values beyond three median absolute deviations, were linearly interpolated. The interpolated values were calculated from neighbouring non-outlier data points for each condition using the MATLAB function *filloutliers* and were implemented as outliers impair the ML performance [62]. Interpolation was selected over data removal to maximise the dataset, as smaller datasets are more prone to overfitting [36]. A total of 4.77 ± 0.49%, 5.16 ± 0.31%, and 4.74 ± 0.15% of the data were interpolated for the model development sample, cross-subject validation sample, and within-subject validation sample, respectively.

After outlier interpolation in MATLAB, all ML processing and analysis were conducted using Python and Scikit-learn [63]. Here, the random seed was set to 123 for all ML analyses. The features for each dataset were scaled to between 0 and 1 and univariate feature selection was conducted. All candidate features were ranked in terms of importance using F-tests and a custom sequential feature selection was implemented. Here, a baseline RF model, with no hyperparameter tuning, was developed with one feature initially. Features were sequentially added, up to a maximum of 100 features (to limit computational complexity), to identify the optimal feature configuration. The optimal number of features for each classification task (scenes—faces; scenes: neutral—pain; and faces: neutral—pain) was defined as the baseline model that achieved the best cross-validation accuracy. Stratified k-fold validation (k = 10) was used as the cross-validation procedure.

Following the identification of the optimal features, the final ML model was developed for each task. Here, a RF model was trained on the model development dataset. Hyperparameter optimisation was achieved using random search, which searches within a range of upper and lower bounds for the optimal hyperparameter values for a user-specified number of iterations [64–66]. The external validation datasets did not inform model development as this can lead to overfitting. Therefore, hyperparameter optimisation was only performed in relation to cross-validation performance. For training and cross-validation, we evaluated model performance using stratified k-fold validation (k = 10) with accuracy as the scoring function. A maximum of 5000 iterations was specified for hyperparameter tuning. Once the optimal hyperparameters were identified, the model was refitted to the entire training dataset. This resulted in the final model that was evaluated using the external validation datasets.

### Model evaluation: discrimination and calibration

The predictive capability of each model was assessed using several performance metrics for each of the validation sets (cross-validation and two external validation datasets). The primary discrimination metrics in this study were the model accuracy and area under the receiver operating characteristics curve (AUC). In addition, we also assessed model performance using alternative metrics including the Brier score, F1 score, precision, and recall. Overviews of these metrics have been reported elsewhere [8, 9, 67–69]. For the external validation datasets, we calculated model performance for each subject and averaged across the entire sample to achieve both individual subject and whole sample accuracies.

In addition to model discrimination performance, we also assessed calibration for models that exceed chance discrimination performance. Prediction algorithms can be subject to bias even when the models demonstrate excellent discrimination performance [70]. Consequently, model calibration, which evaluates the agreement between the model’s predicted probability of an event compared to the reference or observed value, should be assessed [54, 69, 70]. We assessed model calibration using calibration curves for both the cross-subject and within-subject validation sets, segmenting each dataset into 20 bins (see [70]). Calibration curves display the predicted probability on the x-axis and the true probability on the y-axis. Perfect calibration is represented by a 45° line, whereby the predicted and observed probabilities are identical [9]. Calibration has been extensively reviewed elsewhere [70, 71]. Calibration assessment is only necessary when the ML models demonstrate good discrimination ability, as models with poor performance do not require additional calibration assessment [69].

### Statistical thresholding

Theoretically, the chance level for a binary classification task with infinite sample size is 50%. However, sample sizes are not infinite and are often small in neuroscience, resulting in variable chance levels. To quantitatively evaluate whether the ML model significantly outperformed the chance level for each subject, we implemented a statistical thresholding approach based on a binomial cumulative distribution method proposed by Combrisson and Jerbi (2015). The statistical threshold to exceed the chance level can be calculated using the following approach that applies the *binoinv* MATLAB function:

$$Statistical\,Threshold=binoinv\left(1- \alpha , n,\frac{1}{c}\right)* \frac{100}{n}$$

where α is the significance level, *n* is the number of trials per participant, and *c* is the number of classes.

For a given participant with *n* = 200 and *c* = 2, the model accuracy must be above 56%, 58%, and 61% to be significant at the 0.05, 0.01, and 0.001 levels, respectively [72]. If the model accuracy exceeds the given threshold, the performance is significantly greater than the chance level. A minimum of 100 data samples is required to achieve comparable results to permutation testing [72]. For all classification attempts, all subjects had more than 100 trials meaning that the use of binomial testing is acceptable. In all classifications, we use a threshold of p = 0.05. The average chance level for cross-subject and within-subject predictions was 55.20 ± 0.20% and 55.26 ± 0.24%, 57.34 ± 0.37% and 57.41 ± 0.39%, and 57.39 ± 0.36% and 57.24 ± 0.38%, for faces—scenes, scenes: neutral—pain, and faces: neutral—pain classifications, respectively. Finally, to test whether the average sample performance exceeded the average chance threshold for each sample and classification attempt, the individual subject accuracies and chance levels were compared using paired samples t-tests.

## Results

### Self-report ratings

Descriptive statistics of the average self-report pain ratings for each of the four image types across the three samples are presented in Table 1. A 2 × 2 repeated measures ANOVA was conducted using IBM SPSS 27 (IBM Corp., Armonk, New York, USA) to assess the differences between participant pain ratings for the different conditions. The data from samples one (model development) and two (cross-subject validation) were combined for the analysis. There was a significant main effect of image type on the participant’s perceived pain intensity ratings (F (1,90) = 19.89, p < 0.001, η_p_^2^ = 0.18), with the action scene images being rated as more painful than faces. Moreover, there was a significant main effect of pain condition (F (1,90) = 1568.26, p < 0.001, η_p_^2^ = 0.95). Here, the pain condition images received significantly higher pain ratings than the neutral condition images. Additionally, there was a significant interaction between image type and pain condition (F (1,90) = 22.10, p < 0.001, η_p_^2^ = 0.20). Post hoc paired samples t-tests demonstrated that pain ratings were significantly higher in the pain scenes condition when compared to the pain faces condition (t (90) = 4.89, p < 0.001, d = 0.51). There was no significant difference between pain ratings for the neutral faces or scenes conditions (t (90) = 0.68, p = 0.497, d = 0.07). Furthermore, the pain scene images had significantly higher pain ratings when compared to the neutral scene images (t (90) = 38.72, p < 0.001, d = 4.06). Finally, the pain face images received significantly higher pain ratings when compared to the neutral face images (t (90) = 31.09, p < 0.001, d = 3.26).

**Table 1:** Mean ± SD of perceived pain intensity for each condition and sample

Sample	Neutral scenes	Neutral faces	Pain scenes	Pain faces
Development Sample	5.96 ± 8.32	4.87 ± 8.35	61.74 ± 14.04	52.63 ± 18.19
Cross-subject Validation Sample	3.80 ± 3.98	3.93 ± 5.10	63.55 ± 14.49	57.28 ± 14.80
Within-subject Validation Sample	4.87 ± 8.31	4.56 ± 8.91	61.59 ± 10.69	58.38 ± 14.84

### ERP analyses

**Fig. 2:**
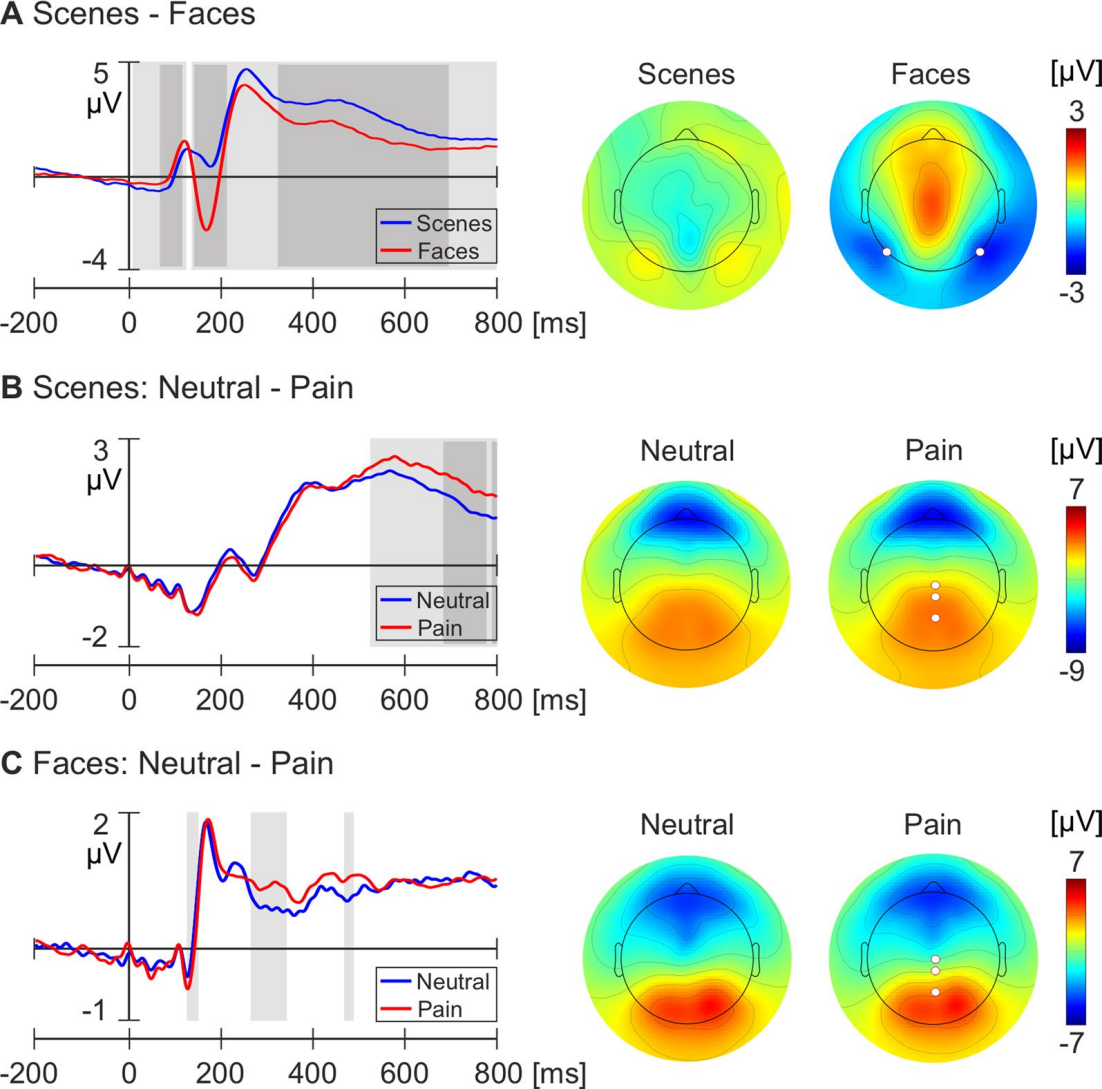
Average ERP waveforms and scalp isopotential maps for each comparison from the unique 91 subjects within samples one and two. **A** Brain responses to scene and face images. Left: Average ERP waveforms from electrodes 58 (P7) and 96 (P8) for each condition. Right: Average scalp potential for each condition between 150 and 190 ms. **B** Brain responses to neutral and pain scenes. Left: Average ERP waveforms from electrodes Cz, 55, and 62 (Pz). Right: Average scalp potential between 524 and 674 ms for each condition. (**C**) Brain responses to neutral and pain face images. Left: Average ERP waveforms at electrodes Cz, 55, and 62 (Pz). Right: Average scalp potential between 270 and 348 ms for each condition. White circles indicate electrode locations of the average ERP waveforms. Light grey bars denote significant differences at p < .05. Dark grey bars represent significant differences at p < .00001

Figure 2A–C show the averaged ERP waveform from select electrodes and the scalp isopotential maps for each condition and comparison (scenes—faces, scenes: neutral—pain, faces: neutral—pain). A significantly stronger negative deflection in response to face images compared to scene images was observed over bilateral occipital-temporal electrodes during the N170 time window (142–214 ms; peak 170 ms; p < 0.00001). Regarding neutral and pain scene images, a significantly stronger positive deflection was observed in a cluster of central-parietal electrodes during the LPP (524–796 ms; p < 0.05), peaking at 578 ms. Similarly, for neutral and pain faces, a significantly enhanced P3 potential (270–348 ms; peak 318 ms; p < 0.05) was observed over central-parietal electrodes in the pain condition relative to the neutral condition.

### Machine learning analyses

**Fig. 3:**
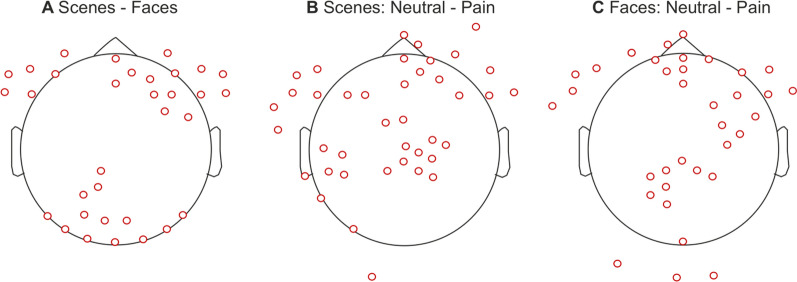
Scalp locations of the important features determined during feature selection and model development for each classification task: scenes—faces (**A**), scenes: neutral—pain (**B**), and faces: neutral—pain (**C**)

Following ERP analyses, the ML analysis was conducted for each of the three classification attempts. From the feature selection procedure, a total of 89, 94, and 90 features were deemed optimal for each classification task, respectively. The scalp locations of the optimal features for each of the different classification paradigms are presented in Fig. 3. Additionally, the number of trials/observations used in the ML analysis for each condition and each sample is presented in Table 2.

**Table 2:** The number of observations/trials per condition and sample used in the ML analysis

Sample	Scenes		Faces		
	Neutral	Pain	Neutral	Pain	Total
Development/Cross-validation (n = 40)	2407	2449	2517	2486	9859
Cross-subject (n = 51)	3156	3151	3194	3176	12,677
Within-subject (n = 25)	1569	1505	1595	1602	6271
Total	7132	7105	7306	7264	28,807

### Faces—scenes classification

**Fig. 4:**
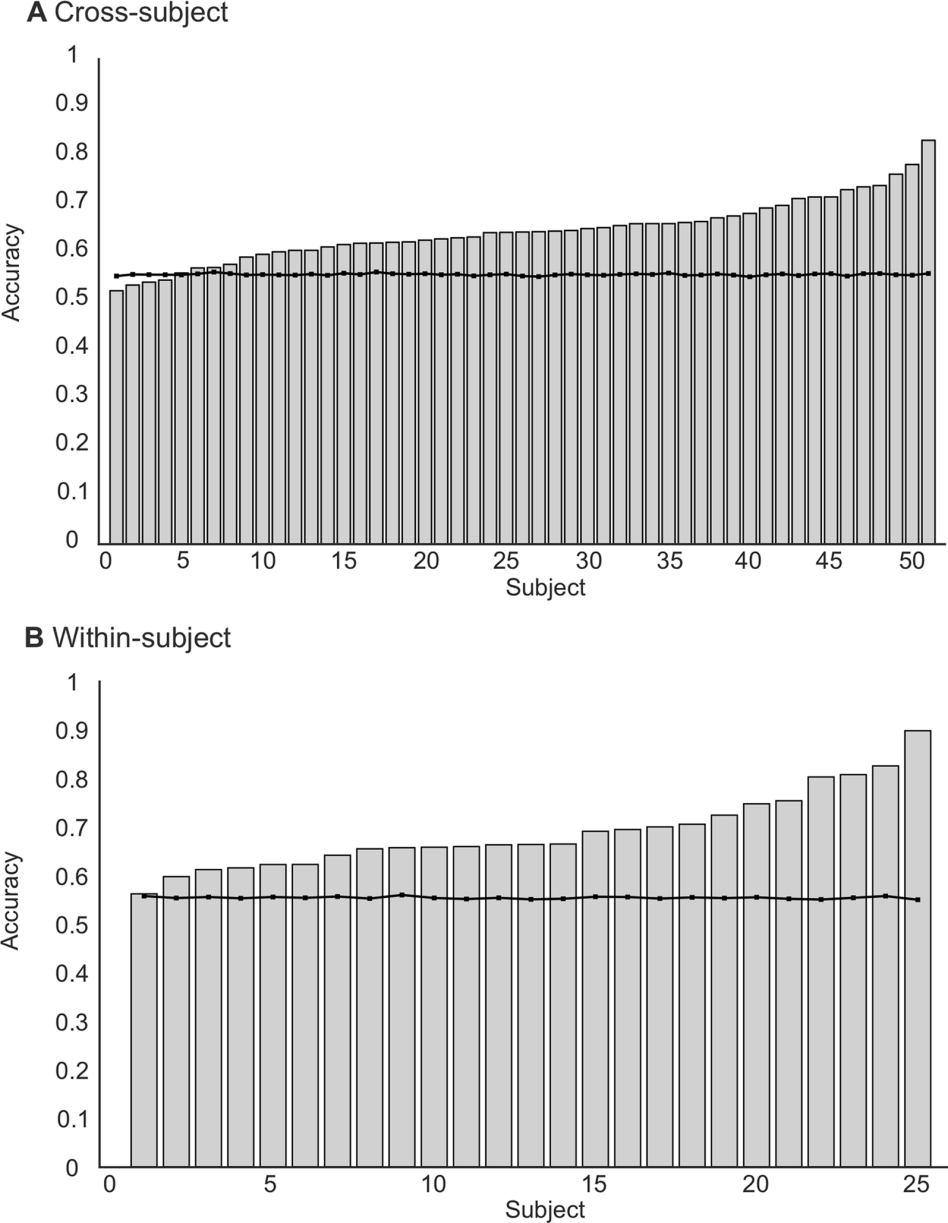
Accuracies for each individual participant for the scenes—faces classification. (**A**) Cross-subject validation dataset. (**B**) Within-subject validation dataset. The black lines denote the significance threshold for chance classification performance at p = .05

The average of each sample’s classification performance metrics and optimal hyperparameters for the classification of face versus scene photographs are reported in Table 3. Additionally, Fig. 4 shows the accuracies and chance thresholds for individual subjects in the cross-subject and within-subject validation samples. The average sample results demonstrate that the RF model achieved an accuracy (± SD) of 0.7456 (0.0459), 0.6415 (0.0634), and 0.6880 (0.0792) on the cross-validation and two external validation sets, respectively. Moreover, the model achieved an average AUC of 0.8189 (0.0406) on cross-validation, 0.7088 (0.0753) on cross-subject validation, and 0.7558 (0.0922) on within-subject validation. Paired samples t-tests demonstrated that the average sample accuracy was significantly greater than chance levels for the cross-subject sample (t (50) = 10.08, p < 0.001, d = 1.41) and the within-subject sample (t (24) = 8.46, p < 0.001, d = 1.69).

**Table 3:** Mean sample performance metrics for scenes—faces classification

Metric	Cross validation		Cross-subject validation		Within-subject validation	
	Mean	SD	Mean	SD	Mean	SD
Accuracy	0.7456	0.0459	0.6415	0.0634	0.6880	0.0792
AUC	0.8189	0.0406	0.7088	0.0753	0.7558	0.0922
Brier Score	0.1707	0.0164	0.2152	0.0253	0.1970	0.0358
F1 Score	0.7854	0.0299	0.6972	0.0460	0.7388	0.0557
Precision	0.6924	0.0495	0.6129	0.0583	0.6597	0.0959
Recall	0.9111	0.0240	0.8207	0.0890	0.8560	0.0802

Optimal hyperparameters: Number of estimators = 766, Maximum depth = 53, Minimum samples to split = 9, Minimum samples at leaf = 2, Maximum features = sqrt, Bootstrap = False

Regarding the individual subject classification performance, the results demonstrate that the model accuracy for 47 of 51 subjects was significantly greater than the chance level (p < 0.05) for the cross-subject validation sample. Moreover, for all participants (25/25) in the within-subject sample, the model achieved accuracies significantly greater than the chance levels.

**Fig. 5:**
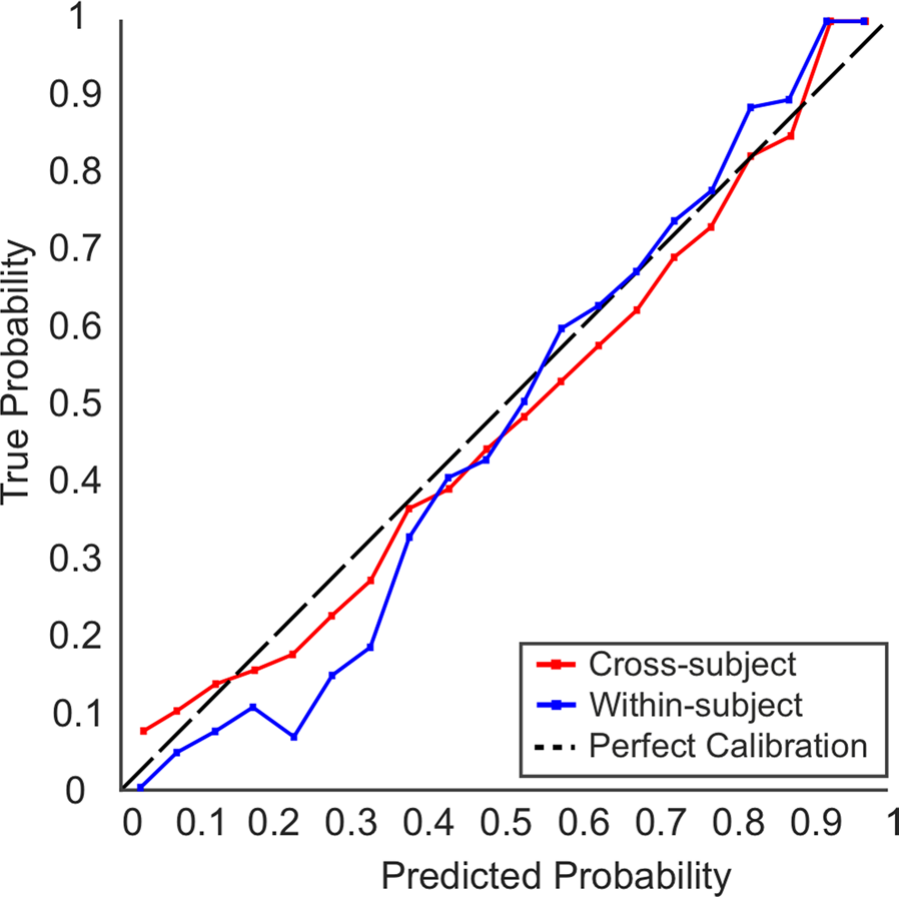
Calibration curves for both cross-subject and within-subject validation datasets for the scenes—faces classification task. The black dotted line (45°) represents perfect calibration

Finally, we also assessed model calibration for the two external validation datasets. The calibration curves for both validation stages are presented in Fig. 5. To interpret the plots, if the model line falls above the reference line it is indicative of underestimating the probability of the outcome, whilst a line below the reference suggests the model is overestimating the probability of the event [9, 70]. The RF model for the faces versus scenes classification task generally demonstrates reasonable calibration for both cross-subject and within-subject datasets. The calibration curves follow the expected trend. Overall, the model is reasonably well-calibrated for both cross-subject and within-subject predictions.

### Scenes: neutral—pain classification

**Fig. 6:**
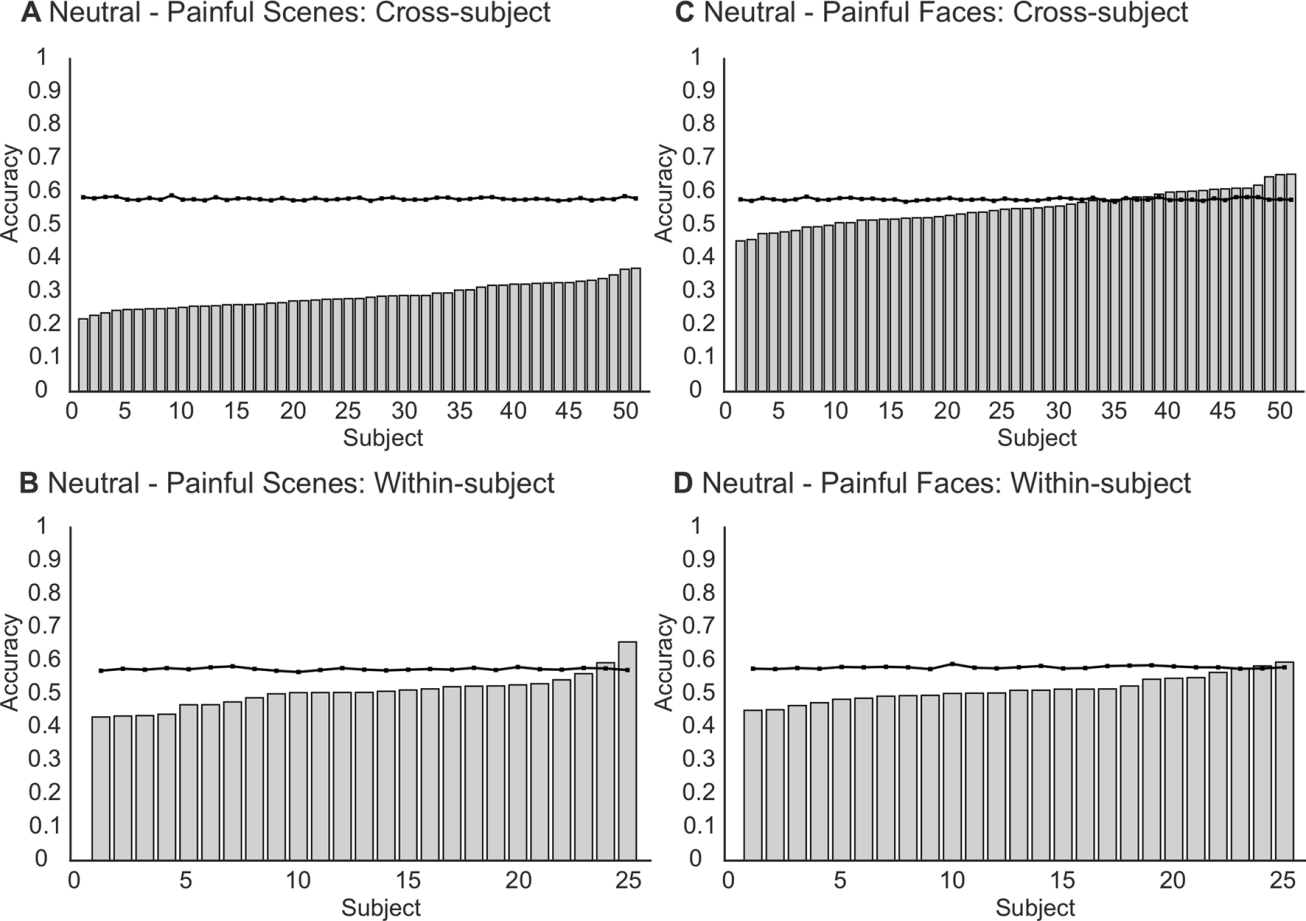
Individual subject accuracies for both cross-subject (top panels) and within-subject (bottom panels) for both scenes: neutral—pain (left panels) and faces: neutral—pain (right panels). The black lines denote the significance threshold for above chance classification performance at p = .05

The average classification performance and optimal hyperparameters for the neutral versus pain scenes classification are reported in Table 4. The average accuracy (SD) was 0.8038 (0.0208), 0.2837 (0.0358), and 0.5065 (0.0504) for cross-validation, cross-subject validation, and within-subject validation, respectively. The AUCs produced a similar trend, with the evaluation procedure demonstrating an AUC of 0.8348 (0.0234), 0.2747 (0.0361), and 0.5123 (0.0518) for the three validation stages. Paired samples t-tests demonstrate that both the cross-subject (t (50) = 57.15, p < 0.001, d = 8.00) and within-subject (t (24) = 6.67, p < 0.001, d = 1.33) performance is significantly lower than the chance threshold. Regarding individual subject performance, the classification accuracy was less than the chance level for all 51 participants of the cross-subject sample. For the within-subject sample, only 2 of the 25 subjects recorded an accuracy significantly greater than the chance level. The results for individual subjects are reported in Fig. 6. Finally, as the models do not outperform chance levels for discrimination, we do not assess calibration.

**Table 4:** Mean sample performance metrics for neutral—pain scenes classification

Metric	Cross validation		Cross-subject validation		Within-subject validation	
	Mean	SD	Mean	SD	Mean	SD
Accuracy	0.8038	0.0208	0.2837	0.0358	0.5065	0.0504
AUC	0.8348	0.0234	0.2747	0.0361	0.5123	0.0518
Brier Score	0.1480	0.0093	0.3966	0.0232	0.3044	0.0257
F1 Score	0.8344	0.0151	0.3866	0.0423	0.4798	0.0554
Precision	0.7277	0.0231	0.3379	0.0340	0.4960	0.0473
Recall	0.9788	0.0204	0.4553	0.0635	0.4682	0.0758

Optimal hyperparameters: Number of estimators = 735, Maximum depth = 46, Minimum samples to split = 28, Minimum samples at leaf = 17, Maximum features = sqrt, Bootstrap = False

### Faces: neutral—pain classification

Finally, the average classification metrics and hyperparameters for the neural and pain faces classification are reported in Table 5. The results demonstrated that the RF model achieved an average accuracy (SD) of 0.6132 (0.0300), 0.5473 (0.0501), and 0.5076 (0.0383) for the cross-validation, cross-subject, and within-subject validation samples, respectively. In terms of AUC, the cross-validation AUC was 0.6717 (0.0396), the cross-subject AUC was 0.5629 (0.0667), and the within-subject AUC was 0.5241 (0.0557). Paired samples t-test indicated that the average sample accuracy was significantly lower than the chance threshold for the cross-subject validation sample (t (50) = 3.82, p < 0.001, d = 0.53) and the within-subject sample (t (24) = 8.57, p < 0.001, d = 1.71). The individual subject accuracies for both the cross and within-subject samples are reported in Fig. 6. Sixteen participants from the cross-subject sample and 2 participants from the within-subject sample achieved classification accuracies significantly greater than chance. As the model performance did not significantly exceed the chance threshold, we do not assess model calibration.

**Table 5:** Mean sample performance metrics for neutral—pain faces classification

Metric	Cross validation		Cross-subject validation		Within-subject validation	
	Mean	SD	Mean	SD	Mean	SD
Accuracy	0.6132	0.0300	0.5473	0.0501	0.5076	0.0383
AUC	0.6717	0.0396	0.5629	0.0667	0.5241	0.0557
Brier Score	0.2268	0.0073	0.2523	0.0155	0.2594	0.0108
F1 Score	0.5944	0.0505	0.5046	0.1053	0.3942	0.1003
Precision	0.6216	0.0353	0.5585	0.0720	0.5182	0.0834
Recall	0.5788	0.0930	0.4932	0.1804	0.3355	0.1200

Optimal hyperparameters: Number of estimators = 161, Maximum depth = 27, Minimum samples to split = 2, Minimum samples at leaf = 4, Maximum features = log2, Bootstrap = False

### Exploratory analysis

As the RF model was unable to significantly exceed the chance thresholds for both neutral and pain scenes and faces classification, we performed exploratory analyses to assess whether a different number of features could improve the classification performance on the external validation datasets. To assess this, we developed and evaluated 100 RF models for each classification attempt, sequentially adding features on each iteration. We initially trained the model with 1 feature and progressed to a maximum of 100 features. The model was then assessed on both validation datasets. The RF was trained using the same procedure as the other models developed in this study, but the number of iterations of hyperparameter optimisation was capped at 500 to reduce computation complexity. The mean, standard deviation, minimum, and maximum values for each of the classification tasks that did not exceed chance performance (scenes: neutral—pain and faces: neutral—pain) are reported in Table 6. The results of the exploratory analysis demonstrated comparable results to the original models developed. Minor performance improvements were observed, however, the model accuracy for both external validation sets remain around the chance classification level.

**Table 6:** Exploratory analysis results (accuracy) for feature combinations (1–100)

Classification	Sample	Mean	SD	Minimum	Maximum
Scenes	Cross-validation	0.7048	0.1155	0.5282	0.8186
	Cross-subject	0.3968	0.1226	0.2689	0.5362
	Within-subject	0.5063	0.0058	0.4889	0.5218
Faces	Cross-validation	0.5978	0.0014	0.5359	0.6128
	Cross-subject	0.5435	0.0063	0.5148	0.5540
	Within-subject	0.5166	0.0068	0.4952	0.5364

## Discussion

We aimed to externally validate and classify single-trial EEG data elicited in response to visual stimuli using ML. Our results demonstrated that the RF model could classify images of scenes and faces with above-chance classification performance for all samples. However, the ML model could not discriminate between neutral and pain depictions of faces or scenes, achieving accuracies comparable to the chance classification rate, or lower. The results support our first hypothesis that the RF model would outperform the chance level for the scenes versus faces classification task. However, the remaining two hypotheses that the RF model would outperform chance for both cross-subject and within-subject samples on both the neutral and pain conditions for face and scene images were not supported as the model performance was significantly lower than chance on all classification attempts. Consequently, the results suggest that large broad category differences (e.g., faces—scenes) are sufficient to achieve above-chance classification performance using external single-trial EEG data. However, more nuanced differences, such as those observed in the neutral–pain classifications, cannot be used to accurately discriminate classes with novel data using the current paradigm.

Our ERP analysis demonstrated an enhanced N170 over bilateral occipital-temporal electrodes in response to face images when compared to scenes, which has been reliably demonstrated previously [24–30]. Moreover, an increased LPP over a cluster of central-parietal electrodes was identified in the pain scene images compared to the neutral condition. Finally, an increased P3 over central-parietal electrodes was observed in response to pain faces compared to neutral expressions. The ERPs elicited in response to the empathic pain processing are also consistent with previous research [22, 23]. Meta-analyses of the ERP components observed during the empathic processing of painful stimuli demonstrated a positive shift in both the P3 and LPP components during the observation of painful stimuli, with the effect maximally observed over the central-parietal region [22]. Therefore, our ERP analysis validates the data quality and experimental paradigm and replicates the effects previously reported in a comparatively large sample of healthy participants.

The findings from this study are comparable and build upon the findings of previous research which demonstrated that discrete categories of visual stimuli could be accurately classified by ML and EEG. We successfully classified images into either faces or scenes, using features predominately located across frontal-central and occipitotemporal regions, which are active during the observation of faces (e.g., N170 and VPP) [24, 25, 31, 32]. Previous research has successfully classified neural responses to visual stimuli including faces, objects, and scenes [1, 3–7]. The present study extends the previous research by externally validating ML and EEG for image classification for both cross and within-subject prediction tasks using a large sample size. Much of the existing literature consisted of small samples (e.g., ≤ 10 subjects) [1–6], which are at higher risk of overfitting, resulting in potentially biased results [36, 73]. Furthermore, previous research did not rigorously assess model performance using external validation, which further increases the risk of poor generalisability [74]. Therefore, the performance and utility of previous models should be interpreted with caution. In addition to generalising to external data, our classification of scenes and faces demonstrated well-calibrated estimates, which provides further evidence of an effective prediction model [70, 71]. Calibration is often omitted in prediction modelling research, but it is essential to evaluating model performance [8, 75]. Consequently, our research provides methodologically superior estimates of the effectiveness of ML and EEG for classifying visual stimuli during passive viewing. To our knowledge, we are the first to externally validate ML models for EEG visual task decoding, providing robust estimates of model discrimination and calibration, and allowing for the interpretation of model generalisability.

The current study demonstrated that ML and EEG were unable to accurately classify neutral or pain faces or scenes. We believe that the low signal-to-noise ratio of EEG and the use of a passive task may have contributed to poor classification performance. Firstly, EEG has a low signal-to-noise ratio which may have resulted in poor discriminative ability for the neutral and pain stimuli classifications [76]. The N170 component offers a distinguishing characteristic between images of face and non-face classes. However, the ERP waveforms for neutral and pain images in either face or scene conditions are similar in their spatio-temporal profile, with differences mainly implicated as enhanced or augmented component fluctuations [22, 23]. Therefore, we can speculate that the differences at the single-trial level may be attenuated by noise and not detectable. Indeed, ML-EEG research often implements spatial filters to improve the signal-to-noise ratio and classification performance [77, 78]. However, we opted against spatial filtering as it has a high risk of overfitting [77, 79]. Alternatively, the improved signal-to-noise ratio of magnetencephalography may allow for improved classification performance [80]*.* Moreover, the use of a passive viewing paradigm may have contributed to the classification performance. Research has demonstrated that passive viewing tasks result in reduced P300 amplitudes when compared to active viewing [81], whilst other component amplitudes (e.g., LPP) are associated with, and altered by, attention and engagement [82–84]. Therefore, any further attenuation of ERPs arising from passive viewing may have hindered the ML algorithm’s ability to detect patterns. Consequently, nuanced differences (such as those elicited due to empathic responses to pain) may not enable accurate classification on the single-trial level during passive viewing. It is possible that active viewing tasks (e.g., requiring image classification performed by the viewer) may improve EEG signal and consequently ML performance. However, requiring input from the subject raises questions about the usefulness of such brain decoding tools, which should preferably allow inferences on behaviour without specific behavioural requirements. Additionally, active viewing may introduce additional confounds, leading to spurious results. Research has demonstrated that stimulus properties could be decoded solely using eye movements in an active viewing task, which was not possible during passive viewing within the same sample [85]. Whilst the impact of active viewing on EEG-ML classification systems should be investigated, it is important to note that, for the method to be genuinely useful and offer novel insight, it should preferably be able to accurately classify responses during passive viewing. Overall, the inability of the ML algorithm to classify neutral and pain images likely stems from poor signal-to-noise ratio and attenuated ERP responses.

Our results highlight the importance of external validation in ML research. Without performing robust, external validation, the generalisability of the ML model cannot be effectively assessed as the results may stem from overfitting [35–37]. Our cross-validation analysis of the pain scenes classification appears promising, with the model achieving an accuracy of approximately 80%. However, by implementing external validation, it was evident that the model was overfitting, achieving an accuracy below the chance level (28%) for the cross-subject dataset and comparable to chance (51%) for the within-subject validation. Therefore, through the external validation protocol, we were able to identify a model with poor generalisability, which may have otherwise been reported as an important finding. Indeed, we are not the first to demonstrate reduced performance when using an external validation [9, 40, 41], which is a significant, but often overlooked consideration when designing applied ML projects. Much of the prediction modelling research (regardless of research domain) does not assess model performance using external validation (e.g., only 5% of prediction modelling articles on PubMed report external validation in the title or abstract) [86]. Caution is advised when reporting or interpreting past ML-EEG results which have only been assessed using internal methods such as cross-validation, as the models are prone to overfitting, resulting in inflated, un-generalisable performance metrics [35, 37, 41]. Overall, our study highlights the importance of robust evaluation procedures when using ML, to minimise the risk of a new replication crisis [87].

The present study has several limitations. Firstly, we used a passive viewing experimental paradigm, which may have resulted in attenuated ERP responses [81]. Whilst we observed significant differences in both the P3 and LPP components in response to neutral and pain images, the differences between the conditions on a single trial level may have not been preserved due to the reduced neural responses associated with passive viewing, the low signal-to-noise ratio, and single-trial variability which may have contributed to poor ML performance [88]. Additionally, informal feedback from participants indicated that the passive viewing task was perceived as ‘boring’, which may have reduced attention, further impacting the neural responses [82–84]. Therefore, passive viewing may not be appropriate to elicit adequate responses that are detectable using ML at the single trial level using the approach outlined in the present study. Future research should implement active viewing paradigms and assess ML performance to build on our findings. For example, a two-alternative forced choice paradigm whereby participants are required to determine the presence or absence of pain may be more suitable for ML classification than passive viewing tasks. Similar forced choice tasks within pain empathy research have been widely reported [22]. Secondly, whilst the images in the study were similar to previous research [23, 46, 48, 49, 89], they may not be extreme enough to be detectable at the single trial level. Future research may wish to explore more intense pain imagery, such as those depicting injury [90], which may elicit larger ERP and behavioural responses. Additionally, the two stimuli categories used in this study (faces and scenes) were not matched for all physical properties (e.g., luminance), which may have confounded the EEG and impacted the classification. Research has demonstrated that properties such as brightness can alter EEG responses [91]. Therefore, we cannot entirely rule out the notion that confounds such as the physical properties of the image contributed to the classification performance. Moreover, we did not record the racial background of the participants in this study. Research has shown that neural responses during pain observation are attenuated when viewing individuals of a different race [92]. Therefore, collecting and reporting the racial background of the subjects in this study could have provided important additional insight. Finally, the current study only recorded neural responses. Future research should aim to record composite measures (e.g., galvanic skin response) to supplement the EEG, which may improve classification performance.

The current study has important significance in the research field. Specifically, we provide the most robust estimates of EEG-ML visual stimuli decoding due to the extensive external validation procedure. We identified a potential limit of ML-EEG techniques, as ML models were unable to accurately classify pain observation above chance levels. However, assuming model performance can be improved, developing an empathy classification tool has important applications in healthcare, such as a supplementary tool for empathy training for healthcare workers [93]. However, performance improvements are imperative before such applications are considered. Currently, we can reasonably predict whether an individual was observing a face or a scene on external data, which represents an important knowledge contribution. However, the criteria typically applied to clinical contexts suggest that models that demonstrate an AUC less than or equal to 0.75 are not deemed practically useful [94]. Given that most of the AUCs in this study do not exceed this threshold, we recommend that improved model performance is pursued to increase the practical significance of the results, with a particular focus on empathic response prediction.

To the best of our knowledge, this is the first study to externally validate ML and EEG for the classification of various classes of visual stimuli including pain or neutral facial expressions and scenes with pain being inflicted on another person, or without pain. Our results demonstrate that ML and EEG can be used to decode neural responses and successfully classify face versus scene images with better-than-chance accuracy. However, the ML models were unable to discriminate between neutral and painful depictions of either face or scene images. Additionally, the ML result questions the suitability of passive viewing tasks for brain-based decoding algorithms. Overall, the study demonstrates promising results for decoding discrete categories of visual stimuli but is unable to identify the observation of pain using single-trial ERP responses. Finally, our results reiterate the importance of robust, external validation procedures to sufficiently evaluate ML-EEG performance; without which may lead to a new wave of impressive, but not replicable, findings.

## Data Availability

The datasets used and/or analysed during the current study are available from the corresponding author on reasonable request.
